# Genomics and transcriptomics to protect rice (*Oryza sativa.* L.) from abiotic stressors: -pathways to achieving zero hunger

**DOI:** 10.3389/fpls.2022.1002596

**Published:** 2022-10-20

**Authors:** Mushtaq Ahmad

**Affiliations:** Visiting Scientist Plant Sciences, University of Nebraska, Lincoln, NE, United States

**Keywords:** rice (*Oryza sativa*, L.), abiotic stress, adaptability, gene, genomics, transcriptomics

## Abstract

More over half of the world’s population depends on rice as a major food crop. Rice (*Oryza sativa* L.) is vulnerable to abiotic challenges including drought, cold, and salinity since it grown in semi-aquatic, tropical, or subtropical settings. Abiotic stress resistance has bred into rice plants since the earliest rice cultivation techniques. Prior to the discovery of the genome, abiotic stress-related genes were identified using forward genetic methods, and abiotic stress-tolerant lines have developed using traditional breeding methods. Dynamic transcriptome expression represents the degree of gene expression in a specific cell, tissue, or organ of an individual organism at a specific point in its growth and development. Transcriptomics can reveal the expression at the entire genome level during stressful conditions from the entire transcriptional level, which can be helpful in understanding the intricate regulatory network relating to the stress tolerance and adaptability of plants. Rice (*Oryza sativa* L.) gene families found comparatively using the reference genome sequences of other plant species, allowing for genome-wide identification. Transcriptomics *via* gene expression profiling which have recently dominated by RNA-seq complements genomic techniques. The identification of numerous important qtl,s genes, promoter elements, transcription factors and miRNAs involved in rice response to abiotic stress was made possible by all of these genomic and transcriptomic techniques. The use of several genomes and transcriptome methodologies to comprehend rice (*Oryza sativa*, L.) ability to withstand abiotic stress have been discussed in this review

## Introduction

**Figure 1:**
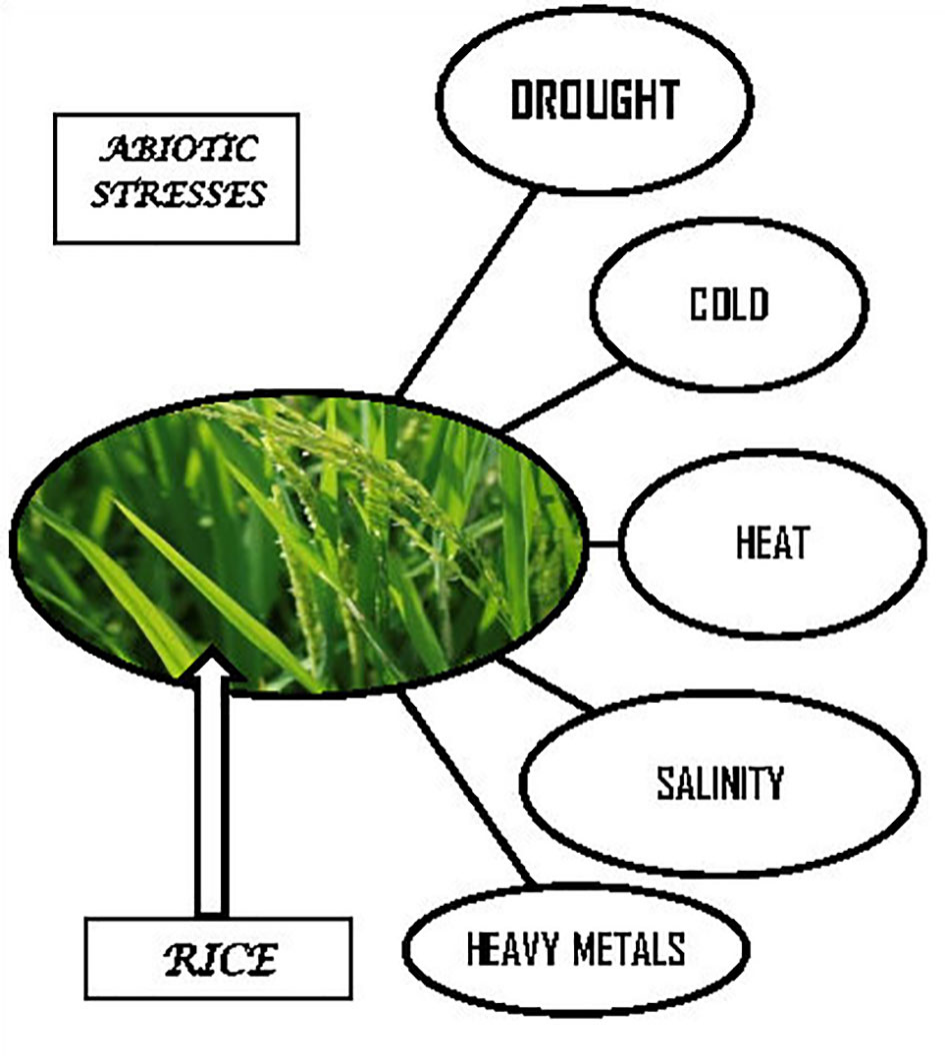
Common abiotic stresses effecting Rice crop.

Adversity is the general term for a variety of environmental challenges that plants frequently face in the natural world and which hinder their survival and development. Abiotic and biotic stresses are the two main types of stress experienced by plants. Physical or chemical factors such high temperatures, droughts, cold injuries; high salt contents, heavy metals, and mechanical damage are the main causes of abiotic stress (**Figure 1**). The main biological causes of biotic stress are fungi, bacteria, viruses, nematodes, and parasitic plants. Plants alter over time at the cellular, organ, physiological, biochemical, and molecular levels to cope with adversity, which has ultimately represented as morphological changes. Following treatment to rice with (200 mM NaCl, 14 days) crop showing salt stress, the chlorophyll a and b contents of rice (*Oryza sativa.* L.) leaves have found to reduced, with the chlorophyll b content of the leaves being more significantly affected (41%) than the chlorophyll a (33%) content. Leaves with low transpiration rates have frequently exposed to high temperatures because heat can cause leaves to heat up quickly (up to 15°C above air temperature). When Na+ and Cl^-^, as well as other cations, are present in the soil in various concentrations (1 to 150 mM for glycophytes; more for halophytes), the roots of plants undergo salt stress. Ion uptake have influenced by a plant’s genotype, growth stage, temperature, relative humidity and light intensity. A surplus of salt in the rhizosphere slows plant growth, reduces yield, and can even kill plants (Hao et al., 2022). The plants benefit from this adaptation to take precautions because it lessens the harm caused by stress. Plant stress has become a significant factor limiting the growth of contemporary agriculture as result of the deterioration of the global environment and research into the plant stress responses mechanism has expanding as well. The chemical mechanism behind the extraordinarily complicated biological process of a plant’s response to stress has not yet fully understood. The term “transcriptome” refers to the collection of all RNAs, including mRNA and noncoding RNA, produced by a particular cell or tissue in a certain functional state. Transcriptomics is the study of gene transcription (type, structure, and function) and regulatory mechanisms in cells at the systemic level. This includes investigating non-coding region function, transcript structure, gene transcription level and novel transcriptional regions.

**Figure 2:**
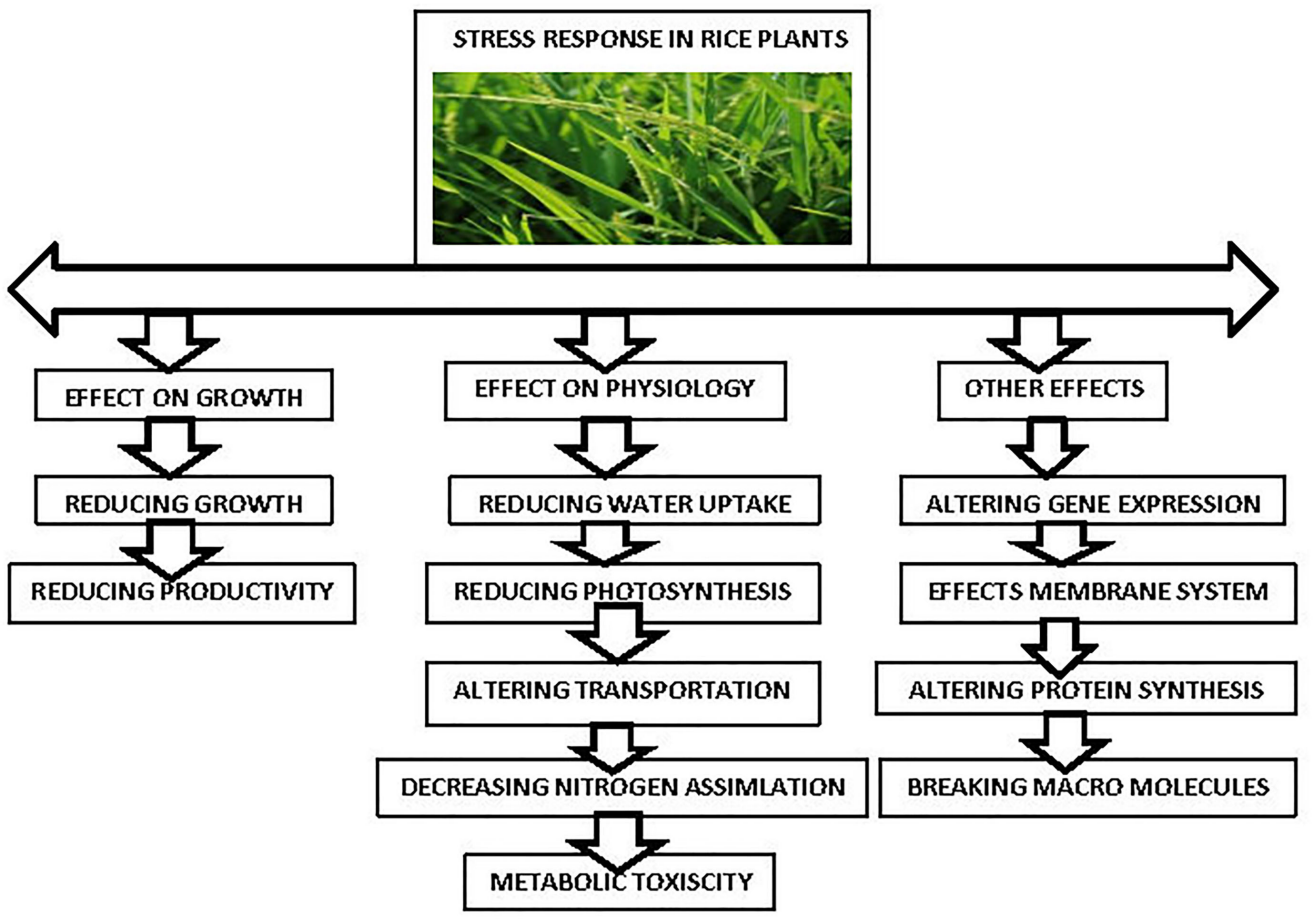
Stress response in Rice crop.

Transcriptomics can be used to identify new genes involved in plant resistance, disclose the intricate regulatory network and expression at the whole genome level under stress, and quantitatively quantify changes in plant gene expression at a specific time point and in a certain condition. The purpose of this study was to review recent developments in rice genomics and transcriptomics studies of plant stress, introduce changes in differentially expressed genes (DEGs) in the metabolic pathways under plant stress **Figure 2**, and look ahead to future research trends to provide a framework for additional plant stress research.

**Figure 3:**
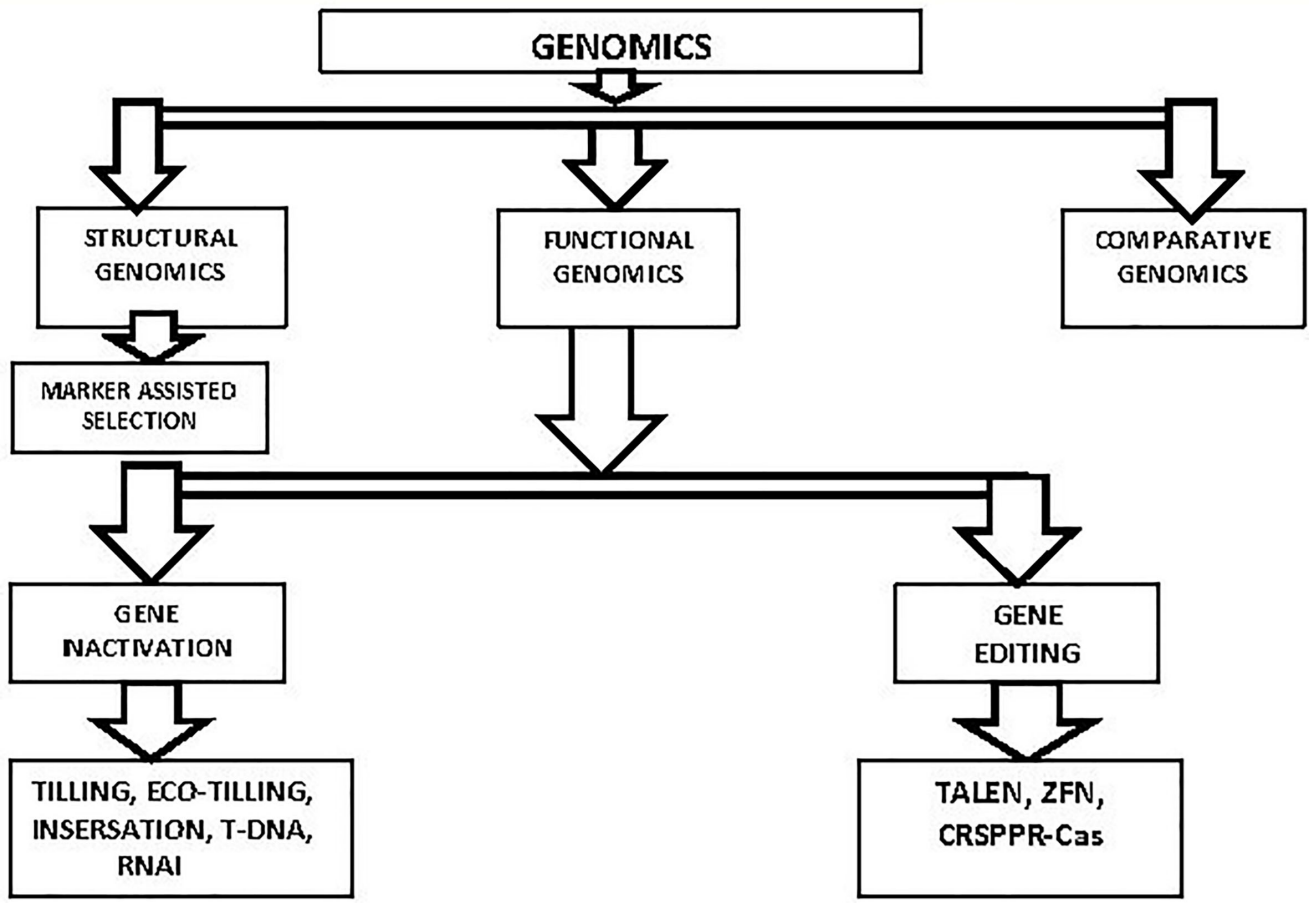
Genomic methods for Rice improvement are. Three main groups of genomic techniques are distinguished: (1) structural genomics, which involves general gene structure and aids in the creation of physical maps; (2) functional genomics, which uses approaches such as gene inactivation and gene editing to identify a gene’s specific function; and (3) comparative genomics, which uses the reference genome sequence of a species to identify gene families in the species of interest.

Most of the world’s population relies on rice as a staple diet, making it a significant crop in terms of agriculture (Deepika and Singh, 2021). With a population of over eight billion people in this century, rice is essential for ensuring global food security (Sasaki and Ashikari, 2018). Similar to other crops, rice is susceptible to unfavorable environmental factors like biotic and abiotic stressors. Pathogens and herbivore assaults are examples of biotic stress, while heat, cold, drought, osmotic, salinity, and heavy metal toxicity are examples of abiotic stress (Grennan, 2006; Zhang et al., 2021). Salinity, temperature swings and drought are abiotic stresses that have an impact on plant production all over the world. This has an impact on productivity, which therefore has an impact on food security. As a result of anthropogenic climate change, which results in more frequent harsh weather, magnifies this adversity (Zhu, 2016). Due to its semi-aquatic and partly radiation environment during evolution, rice has developed a special pattern of abiotic stress susceptibility and tolerance. While other cereal crops may perish in damp environments, rice is tolerant of submersion. However, compared to other grains, rice is extremely sensitive to salinity, vulnerable to drought, and cold (Gregorio et al., 2013). Additionally, because it has grown mostly in tropical and subtropical regions, rice is very susceptible to the chilling stress. As a result, cold stress substantially hinders rice cultivation in temperate regions, especially during the reproductive period (Paul and Roychoudhury, 2019). As a result, rice grown at high latitudes and altitudes is of poor quality (Zhang et al., 2014). Heat stress during reproduction and grain development is particularly harmful to rice (Aghamolki et al., 2014). By the end of the twenty-first century, it has predicted that temperature stress will cause rice yields to drop to 41% (Aghamolki et al., 2014). Two crucial areas need to improve in order to handle these environmental issues: crop management tactics and the creation of elite cultivars. Elite cultivar development has always been accomplished through breeding (Lei et al., 2020). Since the creation of numerous sequencing and bioinformatics technologies, multi-omics methods have now used to create elite cultivars (Roychoudhury et al., 2011). Genomic, transcriptomic, proteomic, and metabolomics methods known as “multi-omics” aid in the discovery and functional analysis of genes, expression analyses, protein-protein interaction studies and the study of their regulatory networks (Rashid et al., 2014). This review goes into detail about the genomic and transcriptomic methods used to identify numerous genes and the regulatory networks that control how rice responds to abiotic stress. Based on the methodology and intended results, genomics has been widely categorized into functional, structural, and comparative genomics (Akpinar et al., 2013). The three categories of genomics, their techniques, and their use in rice for abiotic stress tolerance will covered in this part. **Figure 3** various genetic strategies for rice development is depicted.

## Functional genomics

It has discovered that functional genomic investigations easily yield information useful for crop development. Characterizing how genes function and how they interact with other genes in regulatory networks is a component of functional genomics (Joshi et al., 2019). New genes and their functions has found thanks to recent developments in biotechnological methods. In functional genomics, techniques based on sequencing, hybridization and gene inactivation or editing are all employed (Joshi et al., 2019). Sequence-based techniques include EST (expressed sequence tags), SAGE (serial analysis of gene expression) and 50RACE (rapid amplification of cDNA ends). DNA microarray should have used. Since these methods work with RNA transcripts, they have covered under transcriptomics (Rashid et al., 2014). TILLING (Targeting Induced Local Lesions in Genomes) and T-DNA insertion mutation are the two basic methods based on gene inactivation (Ma et al., 2012). TILLING is a high-throughput method for locating single nucleotide changes brought about by chemically induced mutagenesis in a particular area of a gene of interest. TILLING populations can be used to test for phenotypic and genotypic differences in response to abiotic stressors (Casella et al., 2013). For the purpose of identifying natural polymorphisms, a new technique called EcoTILLING was created (Negrão et al., 2011). Targeted genes are rendered inactive when random T-DNA fragments are inserted into coding or non-coding regions. Targeting candidate genes in plants has shown to be a successful use of T-DNA transformation using Agrobacterium. In addition, gene inactivation and functional research can be done using RNAi technology (Pervaiz et al., 2022).

Genome editing techniques such as targeted mutation, INDEL, and genome-wide sequence changes can be used to characterize the function of plant genes (Rashid et al., 2014). Zinc finger nucleases (ZFNs), transcription activator-like effector nucleases (TALENs) and clustered regularly interspaced short palindromic repeat (CRISPR)-Cas9 (CRISPR associated nuclease 9) are the most frequently utilized genome editing tools (Stephen, 2019: Pervaiz et al., 2022).

ZFNs are nucleases made to cause double-strand breaks (DSB) at particular genomic loci, which results in targeted mutagenesis such as chromosomal deletions, transgene removal and targeted DNA integration (Ran et al., 2018). ZFNs are made up of a *FoKI* type IIS restriction endonuclease’s non-specific cleavage domain coupled to specially made *Cys2-His2* zinc finger proteins and these enzymes produce a DSB. The DNA repair method used by plants is called error-prone non-homologous end joining (NHEJ) (Tsutsui et al., 2011). As an alternative to ZFNs, TALENs came into play; recombinant adeno-associated viruses have used by TALEN to produce specific double-strand breaks and very high success rate for TALEN (Bi and Yang, 2017). CRISPR-Cas9 is now the method of genome editing with the greatest adoption. CRISPRCas9 is an adaptive immune system that fights DNA viral infections in prokaryotes. CRISPR-Cas9, a component of the body’s immune system, has modified to produce an effective genome editing technique. It is made of a synthetic short guide RNA and a Cas9 nuclease that can be modified (sgRNA). Target specificity has provided *via* sgRNA (Bi and Yang, 2017). Consequently, the CRISPR-Cas9 system is simpler to use than ZFNs or TALENs and permits the simultaneous targeting of numerous genes (Zhang et al., 2021).

The effect of RNAi-mediated down-regulation of RACK1, a highly conserved scaffold protein with multiple roles, including plant growth and development was reported by (Li et al., 2009) comparing the transgenic rice to non-transgenic rice plants revealed lower RACK1 expression and resistance to drought stress. The study also showed that transgenic rice (*Oryza sativa.* L.) plants had much higher superoxide dismutase activity than non-transgenic rice (*Oryza sativa*. L.) plants, but membrane peroxidation and malondialdehyde (MDA) generation were significantly reduced (Pervaiz et al., 2022). This implied that rice redox system-related resistance to drought stress negatively regulated by *RACK1*. When the floral meristem specific cytokinin oxidase (*OsCKX2*), gene was silenced in rice using the siRNA method, salt-stress tolerant rice transgenic plants were created without any yield loss (Joshi et al., 2018). Two rice PCS homolog genes, *OsPCS1* and *OsPCS2* have silenced by RNA interference (RNAi) in a recent study, revealing their functions in arsenic tolerance as well (Yamazaki et al., 2018). Transgenic rice with *OsMATE2* from the rice *MATE* family transporter gene was less likely to accumulate harmful arsenic levels (36.9–47.8%) (Das et al., 2018). Summary of abiotic stress-responsive genes in rice identified using genomic approaches **Table 1**

**Table 1:** Summary of abiotic stress-responsive genes in rice identified using genomic approaches.

S. No.	Genes		Function		Associated stress response	Method of validation		References
1	*VP14*		Carotenoid dioxygenase		Drought stress	ECOTILLING		Wang et al. (2004)
2	*OsTP1*		Trehalose-6- phosphate synthase		Drought stress	T-DNA insertional mutation		Kim et al. (2005)
3	*Os05g30750*		Anthranilate phosphoribosyl		Chilling stress	TILLING		Cho et al. (2012)
	*Os12g39210*		Transferase					
	*Os07g36630*		Cyclin					
	*Os01g61160*		CSLF8- cellulose synthase-like family					
	Laccase precursor protein							
4	*OsPIN5*		Auxin transporter		Cold stress	CRISPR-Cas9 mutagenesis		Zeng et al. (2020)
5	*OsAKT1*		Ion transporters		Salt tress	TILLING		Hwang et al. (2016)
	*OsHKT6*			
	*OsNSCC2*			
	*OsHAK11*			
	*OsSOS1*			
6	*SD1*		Plant height		Drought stress	TILLING		Casella et al. (2013)
	*Hd1*		Flowering time	
	*SNAC1*		Drought tolerance	
	*BADH2*		Aroma	
7	*OsCPK17*		Stress signal transduction		Salt stress	ECOTILLING		Negrão et al. (2011))
8	*Os02g0528900*		ABC transporter		Heat stress	TILLING		Hwang et al. (2020))
9	*OsGSK1*		Glycogen synthase kinase		Cold and salt stress	T-DNA insertional mutation		Koh et al. (2007)
10	*OsRR22*		Transcription factor		Salinity stress	CRISPR-Cas9 mutagenesis		Zhang et al. (2019)
11	*OsHSP40*		Heat shock protein		Salt stress	T-DNA insertional mutation		Wang et al. (2019b))
12	*OsCYP19-4*		Cytochrome protein		Cold stress	T-DNA insertional mutation		Yoon et al. (2016))
13	*OsSAP1*		Stress- associated protein		Drought		T-DNA insertional mutation	Giri et al. (2011)	
14	*OsRLCK253*		Receptor-like cytoplasmic kinase						
15	*OsLEA3-2*		Late embryogenesis abundant proteins		Salt and drought stress		T-DNA insertional mutation	Duan and Cai (2012))	
16	*OsDST*		Drought and salt tolerance		Osmotic and salt stress		CRISPR-Cas9 mutagenesis	Santosh Kumar et al. (2020))	
17	*OsMIR528*		MiRNA		Salt stress		CRISPR-Cas9 mutagenesis	Zhou et al. (2017)	
18	*OsTEF1*		Transcription elongation factor		Drought		T-DNA insertional mutation	Paul et al. (2012)	
19	*OsABF2*		ABA- responsive element-binding factor 2		Drought, salinity, and oxidative stress		T-DNA insertional mutation	Hossain et al. (2010)	

## Structural genomics

Functional genomics focuses on the gene, whereas physical genome structure is the emphasis of Structural genomics. The ability to manipulate genes and DNA strands can benefit from understanding the structure of a single genome (Rashid et al., 2014). In structural genomics, high-resolution genetic and physical maps are created. An important method in structural genomics for crop development is molecular mapping and genome sequencing (Chaudhary et al., 2019). Molecular markers are helpful in describing the genetic variety in the germplasm since they has based on the polymorphism seen in any particular DNA (Nogoy et al., 2016). Gene mapping, quantitative trait loci (QTL), germplasm evaluation, and marker-assisted breeding are all common uses of DNA markers in plant breeding (MAB). With the recent advent of genotyping technique based on single nucleotide, polymorphisms have advanced.

## Comparative genomics

The comparative genomics method compares two or more genomes to find out how similar and dissimilar they are, Model plant gene annotations can be applied to freshly sequenced crop species for which functional research has not yet been conducted. For comparative genomics, information on the orthologs that developed from a common ancestor is necessary because these carry out the same function in all species that descended from that ancestor (Wei et al., 2002). In order to find stress-related genes and compare the expression profiles of species, comparative genomics can also use to evaluate the expression profiles of less studied plants under various conditions. The development of phylogenetic trees and multiple sequence alignment are two examples of computational techniques that have used for both intraspecific and interspecific sequence comparisons (Haubold and Wiehe, 2004).

## Functional analysis using gene inactivation and gene editing

Though many polygenes are involved in the regulation of several abiotic stress-related characteristics, these traits are undetectable in single-locus GWAS models. Later on, salt-tolerant loci in rice during the seed germination stage have discovered using multi-locus GWAS techniques. There have found to be 371 QTNs in relation to salt tolerance. In addition, 66 genes have found nearby the 56 QTNs based on functional annotation (Lei et al., 2020). The multi-locus GWAS is therefore highly helpful for identifying salt tolerance genes in rice to identify novel salinity-related candidate genes, characterize the genes and conduct functional investigation *via* overexpression, a number of biotechnological methods have been devised (Lei et al., 2020). The development of next-generation sequencing (NGS) has made it possible to identify thousands of SNPs through genome re-sequencing and the comparison of different genotypes (Lei et al., 2020). Incorporating insertion site-based polymorphisms into molecular markers is a new trend (ISBPs). The polymorphism produced by the insertion regions in the repeat junctions have utilized in this (Nogoy et al., 2016). Chemically altered lines with SNPs in membrane transport genes and variations in how they react to salt stress were found using the TILLING technique. 41 mutant lines with SNPs in the nine target membrane transporters were found among 2961 M2 mutant lines. One study indicated that, 9 out of these genes have exon regions with changed sequences of them, seven could withstand salt stress. Additionally, five mutant lines with SNPs in the exon region of the genes *OsAKT1, OsHKT6, OsNSCC2, OsHAK1* and *OsSOS1* each exhibited different levels of gene expression. These mutations can use to create salt-tolerant lines of plants (Hwang et al., 2020). Similar to this, the Donganbyeo rice cultivar’s TLLING mutant population has screened. These cold-tolerant lines and wild-type plants underwent comparative transcriptome investigations, which revealed that monosaccharide catabolic pathways have related to the resistance to chilling stress. This demonstrates the energy needed for rice to adjust to cold temperatures (Cho et al., 2012). Chalkiness in the grain is a result of high-temperature stress during grain filling, which slows down endosperm production. A multi-omics analysis revealed that α-amylases were upregulated while starch production enzymes have down regulated. According to some research, TILLING mutants of the α-amylases genes may reduce the chalkiness in grains grown under heat stress, obviating the requirement for transgenic lines (Mitsui et al., 2016). Four target genes from TILLING M2 populations of ethyl methane Sulfonate (EMS)-mutagenized lines were validated *(SD1*, *HD1, SNAC1* and *BADH2* involved in determining plant height, flowering time, drought tolerance, and aroma respectively). Since the drop in height shortens the development cycle and triggers drought escape, the two distinct mutations in the *SD1* gene that generated the 21 percent height reduction may aid in drought resistance (Casella et al., 2013). Six abiotic stress treatments have used to analyze the M7 TILLING population made from gamma ray-induced mutations, and a genome-wide association study have used to perform principal component analysis (GWAS). There are two heat-tolerant SNPs at the *Os02g0528900* locus. Future mutational breeding may benefit from these SNPs (Hwang et al., 2020). Eco-TILLING, a strategy that targets genes involved in salt stress signal transduction (*OsCPK17)* or tolerance mechanisms (*SalT)* discovered 15 and 23 SNPs or indels, respectively, among 375 accessions of cultivated rice. According to Negro et al. (2011), it has discovered that these allelic variations are located in the 30-untranslated region (*30-UTR*), which has investigated for salt-tolerant breeding. It was discovered that the group A bZIP transcription factors are crucial for ABA signaling pathways (Banerjee and Roychoudhury, 2017). A homozygous T-DNA insertion mutant of *OsABF2* has created in order to comprehend its function in rice. The mutant was more vulnerable to abiotic stressors such drought, salt, and oxidative stress compared to wild type, and additionally, the mutant was having lower sensitivity for ABA. *OsABF2* was therefore determined to be a stress-responsive gene in rice (Hossain et al., 2010). *OsPP2CA* group members have discovered to be stress-responsive genes in rice. Lines that overexpressed *OsPP108* exhibited greater ABA insensitivity and greater tolerance to drought stress. Additionally, ABA-dependent and ABA-independent signaling pathways interacted in these lines for abiotic stress tolerance, as evidenced by expression profiling employing multiple stress marker genes (Singh et al., 2015).

A total of 11,688 differentially expressed genes (DEGs) were induced by drought and salt stress, including nine materials found in Plantsexpress (accessed on February 1, 2022 at http://plantomics.mind.meiji.ac.jp/OryzaExpress/). There were 6742 DEGs under drought stress, including 2930 up-regulated genes. Salt stress was associated with 7328 DEGs in total, including 3729 up-regulated genes. The heat map of all DEGs showed that some genes’ levels of expression in the leaf or root reduced in comparison to the control during drought or salt stress. Rice has shown to have the abundant late embryogenesis protein *OsLEA3-2*. In comparison to the wild type, the *OsLEA3-2* rice overexpression line created utilizing a binary *pHB* vector performed better under salt stress and was able to resume development after 20 days of abiotic stress treatments (Duan and Cai, 2012). Different plants have discovered to have increased abiotic stress tolerance when exposed to stress-associated proteins (SAPs) that include A20/AN1 zinc finger domains. It was discovered that *OsSAP1* in rice (*Oryza sativa*. L.) interacts with *OsSAP11*, a closely related homolog, and *OsRLCK235*, rice receptor-like cytoplasmic kinase, *via* the A20 domain. Abiotic stress tolerance in rice has boosted by overexpressing *OsSAP11* and *OsRLCK253* (Giri et al., 2011). *CYP5*, an immunophilin protein, has identified to interact with members of ARF guanine nucleotide exchange factors and is required for localization of *PIN1* (auxin efflux carrier). *OsCYP19-4* has discovered to be highly increased in rice under cold stress and to be controlled by stress. Additionally, functional studies using rice that had this protein overexpressed shown that it improved rice grain output and increased resistance to cold stress (Yoon et al., 2016). There has not yet been a lot of genome editing in rice using TALEN and ZFN for abiotic stress tolerance. However, this decade has seen widespread usage of CRISPR-Cas9 for genome editing in rice. *OsMIR408* and *OsMIR528* lines were created using CRISPR-Cas9 knockout mutations of the miRNAs. These lines were salt-sensitive, and it was discovered that these genes were good salt stress regulators (Yan et al., 2011). Numerous salt-related genes has discovered in the last ten years. One of these, *OsRR22*, encodes a 696-amino acid transcription factor known as a B-type response regulator that participates in cytokine signaling. *OsRR22* gene mutation by CRISPR-Cas9 revealed improved salt tolerance. This demonstrates that an increase in salt tolerance is caused by the reduction of *OsRR22* function (Zhang et al., 2019). RNA-Seq analysis revealed that the expression of seven genes, including *LOC Os03g37290*, *LOC Os06g31800, LOC Os09g13440, LOC Os09g19229, LOC Os10g13430, LOC Os10g41040* and *LOC Os12g28177* had been considerably changed (Zhu et al., 2016) *via* controlling the transcription of the downstream genes involved in salt tolerance in plants. Sequence analysis showed that *Os07g0569700 (OsSAP16)* under drought stress had a 1 bp Indel difference between IR36 and Weiguo. The stress-associated protein *OsSAP16* is expressed more when there is a drought (Lei et al., 2020).

Additionally, *OsSAP16*, a candidate gene for *qRSL7*, which controls relative shoot development under salt stress, was found. Accelerator of internode elongation 1 (*ACE1*) and Decelerator of internode elongation 1 (*DEC1)* are two more genes that control stem elongation (Nagai et al., 2020). The *DEC1* gene produces a zinc finger transcription factor (TF) that prevents internode elongation, whereas the *ACE1* gene produces an undetermined function protein that is associated with internode elongation *via* gibberellic acids (GA). Both genes influence cell division induced by gibberellin in the stem node. The gene ACE1C9285 is regulated by SUB1C, a gibberellin-activated transcription factor (TF) whose activity rises in response to submersion (Fukao and Bailey-Serres 2008). SUB1C expression levels seem to be low in cultivars that possess the *SUB1A-1* regulator gene, which is a homolog of SUB1C. Briefly put, rice (*Oryza sativa*. L.) cultivars that express the gene *SUB1A-1* have altered GAs responsiveness, which causes them to use carbon pools for leaf elongation and impede plant growth in general. This adaptability enables them to withstand large floods (Fukao et al., 2006; Xu et al., 2006). However, transcriptome analysis is employed to examine the alterations at the transcript level under diverse environmental or biological contexts (Thompson and Goggin 2006; Stephen, 2019; Pervaiz et al., 2022). Plant annexins are calcium-dependent, multigene families of phospholipid-binding proteins. The OsAnn3 CRISPR-Cas9 knockout mutation demonstrated a cold tolerance phenotype in comparison to the wild type (Shen et al., 2017). One gene discovered through mutation experiments was the drought and salt tolerance (DST) gene, which has shown to be undesired and is present in the genome due to linkage. The DST gene underwent loss-of-function mutation and a 366 bp deletion because of CRISPR-Cas9 genome editing. This mutant line demonstrated better leaf water retention under dehydration stress. These are only a few instances of how functional genomic approaches have applied to analyses rice (*Oryza sativa.* L.) gene expression under abiotic stress. However, many more have been finished throughout time (Yang et al., 2022). The establishment of transgenic or targeted mutant lines for abiotic stress tolerance would be made easier with the use of all this knowledge.

## Molecular mapping and marker-assisted breeding for abiotic stress tolerance

Traditional breeding methods that involve molecular markers are helpful for creating elite lines. The discovery of genotypes with significant structural variation in the genome and the correlation of this variation with stress conditions has made possible through mapping studies. These differences help confirm the target genes for the abiotic stress response (Rashid et al., 2014). Numerous QTLs connected to stress has found. The advancement of accurate NGS technology, DNA polymorphism detection methods, and map-based cloning aid in the identification of more QTLs and the creation of marker sets (Nogoy et al., 2016), 235 accessions of the temperate japonica rice were tested for salinity tolerance, 30,000 SNP markers had already been applied to these accessions. 27 QTLs had been found because of the GWAS analysis. These QTL locations has compared with 300 genes already known to be involved in rice salt tolerance. Numerous QTLs has discovered in close proximity to genes that are involved in calcium signaling and kinases, demonstrating the significance of calcium signaling in the response to salt stress (Frouin et al., 2018). Four potential areas for thousand-grain weight (TGW) under alkali stress have found using bulked segregant analysis-NGS (BSA-Seq). On Chr.2, which had 18 predictive genes, *QTL-qATGW2-2* was located inside a 116 kb region between two molecular markers, *RM13592* and Indel3. Os02g39884 was identified by BSA sequencing as the potential gene for the alkali-tolerance gene locus in rice known as *QTL-qATGW2-2* (Sun et al., 2021). Muthu et al. (2020) goal was to pyramid QTLs in improved white ponni (IWP) for increasing the plant’s capacity to withstand the three stresses of submersion, salinity, and drought. Drought (qDTY1.1; qDTY2.1), salinity (Saltol), and submergence (Sub1) were the QTLs used (Muthu et al., 2020). Numerous molecular markers, or QTLs, haas been established over the past ten years, and these might be used to find rice (*Oryza sativa.* L.) accessions with desired traits and speed up breeding for the creation of new hybrid lines with a variety of abiotic stress tolerance.

## Gene families involved in abiotic stress response

Genome-Wide Analysis Genome-wide gene family analysis and identification are often carried out using sequence homology of known genes found in a novel crop genome. This results in almost all of the members of that specific gene family being identified. Additional expression analysis aids in the discovery of a powerful gene family member participating in a certain function and aids in the discovery of pseudogenes (Haubold and Wiehe, 2004). Numerous gene families has found in the rice genome using this method. Pentatricopeptide repeat proteins (*PPRs*) are motifs made up of 35 amino acids that have the ability to bind RNA strands after transcription and take part in RNA processing. 491 *PPR* genes discovered in rice (*Oryza sativa.* L.) by genome-wide research, of which *246 PPR* genes belong to subclass P and 245 genes to subclass *PLS*, 7 of these PPR genes were strongly activated under salinity and drought, according to expression analyses, which revealed that numerous PPR genes were induced under biotic and abiotic conditions (Chen et al., 2018). A comparative investigation of 11 rice (*Oryza sativa.* L.) species has conducted to examine the evolutionary and conservation pattern of dehydrin in rice (*Oryza sativa.* L.). Three DHNs were determined to be highly conserved out of the *65 DHNs* that were detected. Dehydrin gene conservation and patterns of domestication and diversification were studied for their association. This demonstrated that while domesticated species like *Oryza nivara* and *Oryza sativa* spp. indica display a conserved evolutionary pattern, wild species like *Oryza rufipogon* and *Oryza sativa* ssp. japonica follow an adaptive evolutionary pattern (beneficial genes getting selected against deleterious alleles) suggesting diversification (Verma et al., 2017). *DUF221* domain-containing genes (DDP genes) are known to play significant roles in stress responses, hormone signaling pathways, and plant growth. Comparative genomics has revealed that both farmed and wild rice (*Oryza sativa.* L.) contains at least nine DDP gene members. *OsDDP6* was upregulated at all stages of development in FL478, salt-tolerant rice genotype despite several expression investigations demonstrating that they are upregulated by salt stress (Ganie et al., 2017).

A key secondary messenger, Ca2+, participates in several signaling pathways that activated by stress. Rice has a diverse set of Ca2+ transport genes that has found using genomic techniques. Utilizing microarray and qRT-PCR techniques, their expression pattern was investigated during several vegetative and reproductive developmental phases, including seedling, mature leaf, panicle, and seed developments (Singh et al., 2014). By taking part in the ribosome biogenesis process, ribosomal proteins (RPs) contribute to translation. It has previously discovered that both biotic and abiotic stress responses include the ribosomal protein large subunit (RPL). 56 ribosomal protein small subunits were found in the rice genome, according to genome-wide investigations (RPS). It has discovered that all 56 *RPS*, evenly distributed among the 12 chromosomes. *RPS* genes have found to implicate in both biotic and abiotic stress responses, according to expression analyses. Under the majority of the abiotic stressors, RPS4, 13a, 18a, and 4a have demonstrated to have greater transcript levels (Saha et al., 2017). It has discovered that proteins from the Dirigent (DIR) and DIR-like family are involved in lignification. 49 DIR or DIR-like genes were found in rice as a consequence of the genome-wide study Of 49, 23 DIR or DIR-like genes are discovered to be involved in the response to abiotic stress (Liao et al., 2017). Phospholipase A (Singh et al., 2012a), MADS-box family (Arora et al., 2007), phytocyanin (Ma et al., 2011), BURP (Ding et al., 2009), armadillo (Sharma et al., 2014) and arabinogalactan (Ma and Zhao, 2010) are examples of other gene families that include members, Nuclear factor Y (Yang et al., 2017); ABA repressor (ABR1) (Mishra et al., 2013);

transcription factors; *NAC*, *ZF-HD*, *WRKY*, *EREBP* and *bHLH* (Muthuramalingam et al., 2018); phospholipase C (Singh et al., 2013); autophagy-associated genes (*ATG*);Transcriptional regulation of gene expression is a component of a plant’s response to stress, and it is dependent on a number of transcription factors and how they interact with the promoter region of the genes. Finding stress-inducible promoter regions is crucial (Roychoudhury et al., 2008). This knowledge may help with the deployment of transgenes with certain stress-inducible promoters (Cohen et al., 2017). Three stress-responsive genes *OsABA2*, which codes for zeaxanthin epoxidase, rab16A, which codes for dehydrin, and HP1, which codes for an unidentified protein—had their promoter elements chosen by Rai et al. (2009), inserted into the rice plant system coupled to the gusA reporter, and it was discovered that the *OsABA2* promoter was the most efficient one. Because it demonstrated that a transgene expressed weakly constitutively under normal circumstances and strongly under abiotic stress (Rai et al., 2009). Cis-acting promoter elements involved in the expression of genes that respond to cold and dehydration have thoroughly studied in Arabidopsis. The same has not done with rice (*Oryza sativa.* L.), though; three different rice species’ transcription patterns for cold and dehydration were created in order to achieve the same goal. Additionally, all three species shared similar responsive genes. The conserved regions in these genes’ promoters that have induced by cold and dehydration revealed that they contain the abscisic acid-responsive element (ABRE) and were the most responsive to dehydration in all three species. Additionally, the sequences *CGTACG* and *GTAGTA*, has found to be novel cold-inducible rice promoters (Maruyama et al., 2012). Aluminum detoxification in rice mediated by a transcription factor known as *ART1* (AL RESISTANCE TRANSCRIPTION FACTOR1). The 31 downtown genes have controlled by ART1, a zinc finger transcription factor of the C2H2 type. These genes’ promoter regions were examined, and it was discovered that *GGN(T/g/a/C)V(C/A/g)S(C/G)* functions as a cis-acting element to cause an aluminium poisoning response (Tsutsui et al., 2011). In rice and Arabidopsis, the distribution and spatial patterns of 2 cis-regulatory elements, *ABRE* and *CE3* has investigated. In contrast to *CE3*, it has discovered that ABRE is equally common in both rice and Arabidopsis (Roychoudhury et al., 2008). The *ABRE* element has organized as an *ABRE-ABRE* pair and creates complexes that respond to ABA. *ABRE* and *CE3* were also present in numerous additional unique combinations in rice gene promoters (Gómez-Porras et al., 2007). Using comparative genomic techniques, numerous more gene families have described at the genome-wide level in rice in addition to these genes and promoter sequences. All of this knowledge will be useful for future functional and structural genomic approaches to the development of novel kinds.

## Transcriptomics methods for rice abiotic stress tolerance

The collection of all RNAs produced by the cell or tissue in a specific functional state is known as the transcriptome. While the name “transcriptomics” refers to the study of the type, structure, function, and regulation of gene transcription, this covers both coding and non-coding RNA (Nejat et al., 2018). Transcriptomics can be used to quantitatively analyze the variations in plant gene expression at a particular time point and state during stressful conditions; this aids in understanding the regulatory network and expression at the whole genome level and identifies new genes related to stress tolerance and adaptability (Wang et al., 2020). Since the advancement of transcript sequencing and analysis technology, transcriptomics has reached its pinnacle. Only single transcripts or small groups of transcripts may be studied at a particular time using traditional transcriptomic methods like northern blotting and RT-PCR (Roychoudhury and Banerjee, 2015; Lowe et al., 2017). Transcript profiling underwent a revolution when microarrays entered clinical use in the middle of the 1990s. This enables the simultaneous investigation of thousands of genes on a massive scale (Wang et al., 2020). Later, the real-time RT-PCR or qRT-PCR technology developed into a very sensitive approach for the detection of transcripts with low abundance. It has been frequently utilised for relative quantification of many gene expressions or absolute quantification of a certain gene (Nejat et al., 2018). For the second time recently, NGS has essentially changed gene expression profiling. High-throughput sequencing enhanced our understanding of epigenetics and gene regulation networks. The discovery and quantification of known unique and less common transcripts, including coding and non-coding RNA, are made possible by NGS-based RNA sequencing (RNA-Seq) (Lowe et al., 2017).

## Technologies in transcriptomics

**Figure 4:**
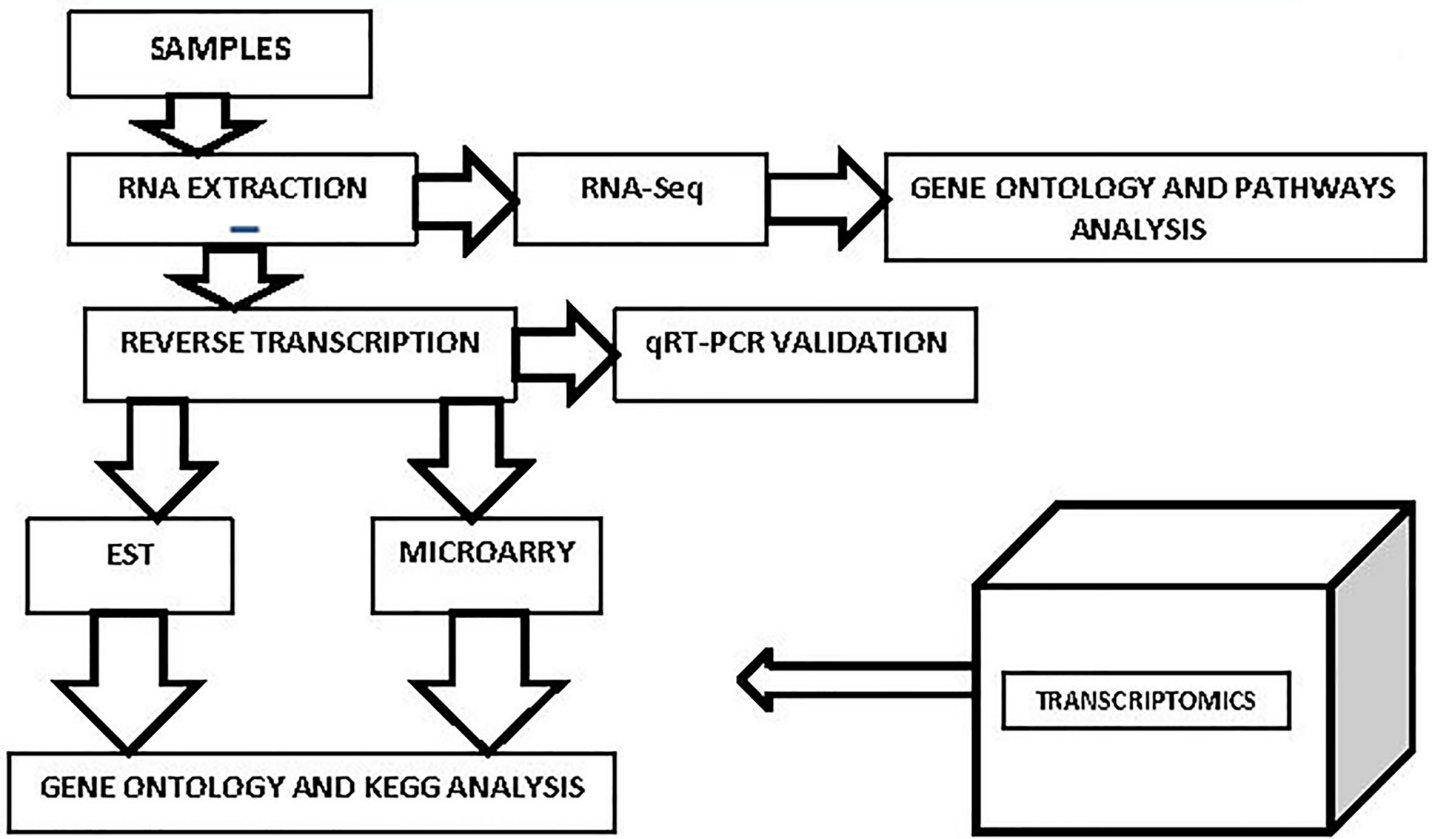
Workflow for transcriptomic techniques for crop improvement Transcriptomics entails the extraction of an organism’s total RNA at a specific time point and under a specific environmental situation, which is then processed differently for expression analysis. Reverse transcriptase is used to create cDNAs for EST and microarray research, and cDNA libraries are then created. For the purpose of creating ESTs and microarray probes for expression profiling, this might be sequenced. If not, the total RNA could be normalised and sequenced in order to identify all the genes with differential expression. qRT-PCR offers additional validation. KEGG analysis and gene ontology aid in the functional connection of discovered genes.

Individual transcripts at random called ESTs are sequenced from cDNA libraries. One-time, low-throughput Sanger sequencing of ESTs was once thought to be a highly effective way to determine an organism’s gene composition without sequencing its complete genome (Nejat et al., 2018). Serial analysis of gene expression, a type of sequencing-based gene expression study that followed ESTs, has developed in 1995 (SAGE). This comprises concatenated random transcript segments that have undergone sanger sequencing. By comparing the transcripts with recognized genes, the quantification has completed. Digital gene expression analysis, a SAGE version utilizing high-throughput sequencing methods, was also briefly used (Lowe et al., 2017). Modern methods like RNA-Seq and microarrays have surpassed these ones (Wang et al., 2020). **Figure 4** depicts the process of transcriptomic methods for the identification of genes. EST can be used to determine which genes an organism expresses at a certain moment. EST can be used to determine which genes an organism expresses at a certain moment. A cloned cDNA is sequenced once to produce an EST. The first step in EST production is the extraction of mRNA from an entire organism or a selected set of tissues (Lowe et al., 2017). Libraries of cDNA are produced by reverse transcription of mRNAs. Random colonies are put through a single sequencing procedure with universal primers. Following sequencing, the raw sequence reads are processed using bioinformatics techniques to get rid of contaminated vector sequences and low-quality sequences. Following that, the generated sequences are added to the dbEST database (Parkinson and Blaxter, 2009; Meng et al., 2010). In addition to supporting complete genome sequencing and gene discovery, EST has its applications.

ESTs are unaffected by probe selection and hybridization intensity, unlike microarray. However, according to Parkinson and Blaxter (2009), ESTs can offer sequence data for creating new microarray platforms. The “probes” that make up microarrays are small nucleotide oligomers that are bonded in an arrayed pattern on a solid substrate. The determination of transcript abundance of the transcripts is used for the expression analysis. It happens when the fluorescently tagged transcript has hybridized to these probes. Each probe locus’ fluorescence intensity on the array corresponds to the amount of transcripts for that probe sequence (Lowe et al., 2017). High-throughput sequencing and computational techniques have coupled in RNA-Seq to determine the number of transcripts in an RNA pool. Depending on the sequencing technique used, the nucleotide sequences produced can range in length from about 100 bp to more. A reference genome or another transcript is used to align the RNA transcripts. RNA-Seq is helpful in identifying genes within a genome or determining which genes are active at a specific period, and it may precisely determine the relative gene expression level (Fujii et al., 2011; Garg and Jaiswal, 2016; Nejat et al., 2018).

## EST and microarrays for rice abiotic stress-related gene identification

Gorantla et al. (2007) discovered genes linked to rice (*Oryza sativa.* L.) sensitivity to water stress. The discovery of 5815 rice ESTs was made possible through the analysis of 7794 cDNA sequences. In the public databases of rice farmed under normal conditions, 334 of these revealed no sequence homology with any rice ESTs or full-length cDNAs; this suggests that these transcripts have enriched during drought stress. A study of these ESTs revealed that 1677 of them contained unique sequences. This sequence has contrasted with abiotic stress-induced sequences from Arabidopsis, barley, maize, and rice expression profiling. 589 potential stress sensitive genes were discovered as a result of this analysis. These have once more compared to the expression profile of the panicle library during drought stress, and these revealed 125 genes out of 589 has expressed in both the panicle and leaf tissues under drought. These 125 genes’ gene ontologies revealed that the majority of them have connected to cellular metabolism, signal transduction, and transcriptional control (Zhou et al., 2008; Gorantla et al., 2007). Similar to this, numerous distinct gene families in charge of abiotic stress in rice have been discovered over time utilising ESTs and microarray approaches. Here, a handful of them are covered. It was discovered that rice (*Oryza sativa.* L.) ability to withstand biotic, abiotic, and arsenate stress depends on glutathione S-transferases (GSTs). GSTs were found to play overlapping and distinct roles in rice during different developmental stages and to mediate cross-talk across multiple stress and hormone response pathways, according to transcript profiling utilizing microarray and ESTs (Jain et al., 2010; Sato et al., 2013). Plants respond to heat stress by producing heat shock proteins, which has then activated by heat shock factors (HSFs). According to Andrási et al., 2021, there are three classifications of plant *HSFs*: A, B, and C. Eight *OsHSTs* are increased during seed development and six HSFs are upregulated during abiotic stress in both the root and shoot, according to expression profiling research using ESTs, microarrays, and qRT-PCR. Stress from cold and dryness results in an up regulation of OsHSFA2a and OsHSFA3. When OsHSFB4a’s expression was profiled using FL-cDNA/ESTs and qRT-PCR, it exhibited either no change or a very little change (Chauhan et al., 2011). Low molecular weight, cysteine-rich metal-binding proteins have known as metallothioneins (MT). The rice genome sequence’s bioinformatics analysis identified 13 genes and 15 protein products (Banerjee and Roychoudhury, 2021). The cysteine amino acid has preserved in the *OsMT1e-P* protein and it was discovered that this contributed to salinity stress. *OsMT1e-P* was abundant throughout seedling and reproductive phases, which are stress-sensitive stages, according to the EST database and publically available microarray data (Gautam et al., 2012). In dicot model plants Arabidopsis thaliana, half-size adenosine triphosphate-binding cassette transporter subgroup G (ABCG) genes were discovered to play a function in abiotic stress, however, this was not confirmed for rice (*Oryza sativa*. L.). Using *FL-cDNA* and EST databases, 30 half-size *ABCGs* in rice were discovered, and their preliminary proof of gene expression under abiotic stress was established. The expression of *OsABCG2*, 6, 10, 24, and 29 has not shown to exist. Under semi-quantitative RT-PCR, the expressions for *OsABCG6* and *OsABCG10* have not found. It was inferred from this that these two genes are psuedogenes (Matsuda et al., 2012). Being a model plant and significant agricultural crop, rice is a C3 plant and hence a prospective candidate for genetic engineering of the C4 pathway. It is already known that C3 plants contain the genes necessary to make C4 enzymes. Through sequence homology, 15 genes from the rice C4 gene families have discovered in the rice genome using the maize C4 gene sequence as a query (Hamada et al., 2017; Jiang et al., 2019; Muthusamy et al., 2019). All of the identified genes had at least one EST or FL-cDNA, according to the results of the expression study utilizing the EST and FL-cDNA databases. Osnadp-me2 and Osnadp-me3 have found to be elevated under salt and drought stress employing microarray datasets for abiotic stress and heavy metal-regulated expression analyses. During salt, drought, and anoxic environments, *Osppdk1* was increased (Muthusamy et al., 2019). The protein breakdown process and the ubiquitination of proteins are both mediated by the ubiquitin-conjugating enzyme E2s (UBCs) in rice (*Oryza sativa.* L.), 39 UBC genes were discovered. EST, microarray and qRT-PCR-based *OsUBC* gene expression profiling revealed that many of these genes had widespread and tissue-specific expression patterns. Furthermore, it has discovered that 14 *OsUBCs* showed differential expression in treatments with salt or drought conditions (Singh et al., 2012b; Zhiguo et al., 2015). A microarray-based analysis of rice seedlings’ salt-induced genes revealed 1834 genes to be elevated by salt stress. One of these revealed a 23.3-fold induction in an EST. Database searches revealed that this EST encodes a type 2C protein phosphatase that was previously unidentified called *Oryza\sativa* salt-induced *PP2C* protein 1 (*OsSIPP2C1*) (Li et al., 2013). Cold induced *MYB1* is a potential MYB transcription factor that was discovered through analysis of the cold-induced transcriptome (*CMYB1*). The expression of *CMYB1* rose more than 100-fold during cold stress, according to qRT-PCR analysis. Additionally, it was discovered that *CMYB1* contributes to circadian rhythm (Duan et al., 2014). It has established that the protein MDCP, which contains the Meprin and TRAF homology (MATH) domain, participates in the response to biotic stress. *OsM4* and *OsMB11* are engaged drought and salinity stress out of the 11 MDCP in rice according to expression analysis utilizing qRT-PCR and microarray (Kushwaha et al., 2016). Similar to this, several genes in rice that respond to abiotic stress have been discovered using EST and microarray techniques. EST libraries have uploaded to the internet for public use in the future. Two sizable EST databases, dbEST and UniGene, both from NCBI, provide EST information from a range of organisms (Singh et al., 2019). There are also numerous EST databases dedicated to rice (*Oryza sativa*. L.) viz, OryGenesDB (Droc et al., 2006), rice Genome Knowledgebase (RGKbase) and the rice Genome Annotation Project (Tanaka et al., 2008: Wang et al., 2013). The rice Expression Profile Database rice (*Oryza sativa.* L.) (XPro) is a collection of gene expression profiles obtained using microarray analysis, and it may contain data from all growth stages, tissues, and stressors (Sato et al., 2013). Rice Oligonucleotide Array Database (ROAD) (Cao et al., 2012), Rice ArrayNet (Lee et al., 2009), Rice MetaSys (Sandhu et al., 2017) and Oryza Express are more rice microarray databases (Hamada et al., 2011; Bandurska, 2022).

## Rice RNA-seq for abiotic stress-related gene identification

Expression analysis utilizing RNA-Seq has reached its pinnacle in the last 5–10 years. The few abiotic stress-responsive genes discovered by RNA-Seq are listed in **Table 2**. Two inbred indica lines, Apo, a moderately drought-sensitive line, and IR64, a drought-sensitive line, were analysed using RNA-Seq to reveal that IR64 underwent transcriptional alterations linked to signal transduction, protein binding, and receptor function (Ereful et al., 2020). Genes connected to an oxygen-binding function and the peroxisome pathway has higher expression levels. The scientists concluded that drought-tolerant cultivars use energy-efficient routes as a reaction to drought while drought-sensitive lines use energy-consuming pathways, failing to live as tolerant plants (Ereful et al., 2020). In order to reduce stress during germination and ingestation, seeds turn on a number of genes. In initial imbibition of rice seed germination, genes related to the cell wall, abiotic stress, and antioxidant-related DEGs were associated with stress response, according to RNA-Seq analysis between 8 h of imbibed seeds and dry seeds of rice (Zhao et al., 2020). Pectin esterase and polygalacturonase were major genes connected to cell walls, while receptor kinase and pectin esterase were prominent genes associated to signaling. Significant abiotic stress-related genes included those with the cup in domain protein, methyl transferases, SPX domain, GSTs, and peroxidase. At that moment, qRT-PCR analysis revealed GST to have the maximum activity. GSTs may stop the early imbibition stage’s burst of H2O2 buildup, which is a factor in the subsequent successful seed germination (Zhao et al., 2020). To deal with the water shortage and to assure a sustainable production in the future, rice needs to be adapted to the aerobic condition. When comparing cultivars that were aerobically adapted (drought tolerant) and those that were anaerobically adapted (drought intolerant), RNA-Seq analysis of the root and shoot revealed that the number of differentially expressed transcripts was higher in the root than it was in the shoot in both aerobic and anaerobic conditions (Phule et al., 2019). Additionally, it was revealed that anaerobic cultivars lacked the high expression of MADS transcription factors and transporters involved in sugar (*SWEET3A*) and nutrient uptake. This implies that these genes are crucial for the ability to withstand drought (Phule et al., 2019). 56 differentially expressed genes were discovered in growing seeds during combined heat and drought stress using RNA-Seq analysis of Aus, drought, and heat-tolerant cultivars. *B12288*, one of the significantly induced genes, has *RAB21* as its annotation. Different *O. sativa* subspecies and various wild species of *Oryza* include homologs of this gene. It is a member of the LEA protein family known as dehydrin (Roychoudhury et al., 2007). Other rice cultivars also included the *RAB21* gene. Despite the small sequence variations, the functions were different (Jing et al., 2020; Schaarschmidt et al., 2020). It is yet unknown how dehydrin causes plants to respond positively to stress control. Recent research has revealed that in Arabidopsis, a change of four amino acids in the LEA protein increased membrane stability under cold stress. (Shankar et al., 2016). Therefore, it was determined that even little changes in the amino acid sequence of RAB21 from various species of rice have a big functional impact (Schaarschmidt et al., 2020). Using RNA-Seq for comparative study, it has discovered that members of the NAC and DBP transcription factors have differentially regulated under salt and dehydration stress in the drought- and salt-tolerant cultivars N22 and Pokkali, respectively (Shankar et al., 2016; Yuan et al., 2021). While transcripts involved in wax and terpenoid metabolism were upregulated in Pokkali, transcripts encoding thioredoxin and those related in phenylpropanoid metabolism were upregulated in N22. This demonstrates how rice plants respond to varying levels of abiotic stress through a variety of transcription factors and metabolic pathways.

**Table 2:** Summary of abiotic stress-responsive genes in rice identified using transcriptomic approaches.

S. No.	Genes	Function	Associated stress response	Method of validation	References
1	*OsMT1e-P*	Metallothioneins	Drought and salinity stress	Microarray	Gautam et al. (2012)
2	*OsUBCs*	Ubiquitin- conjugating enzymes	Salt and drought stress	EST, Microarray	Zhiguo et al. (2015)
3	*OsHsfA3*	Heat shock factor	Heat stress	EST, Microarray	Chauhan et al. (2011)
	*OsHsfA7*				
	*OsHsfA9*				
	*OsHsfA1*				
4	*Os01g47050*, *Os01g59690*, *Os02g15950*, *Os02g51350*, *Os04g33820*, *Os05g43490*, *Os06g39370*, *Os07g09710*, *Os10g30280*	F-box proteins	Osmotic stress	EST, Microarray	Jain et al. (2007)
5	*OsABCG26*	Half-ABC proteins	Cold stress	EST	Matsuda et al. (2012)
	*OsABCG27*		Salt stress		
6	*OsDhn1*	Dehydrins	Drought stress	EST	Lee et al. (2005)
7	*Os02g47744*, *Os12g41920 Os06g19980*	MYB transcription factors	Drought stress	EST, Microarray	Katiyar et al. (2012)
8	*OsZFP177*, *OsZFP181*, *OsZFP176*	A20/AN1-type zinc finger protein	Drought, cold, and osmotic stress	EST, Microarray	Huang et al. (2008)
	*OsZFP173*				
	*OsZFP181*				
	*OsZFP176*				
	*OsZFP157*				
9	*OsDHOD1*	Dihydroorotate dehydrogenase	Salt, drought	EST, Microarray	Liu et al. (2009)
10	*OsSAP16*	Stress- associated protein	Drought stress	RNA-Seq	Lei et al. (2020)
11	*OsHPPD*	Vitamin E biosynthetic enzymes	Dehydration, cold, and salt stress	EST, Microarray	Chaudhary and Khurana (2009)
	*OsyTMT*				
	*OsHPT*				
	*OsMPBQ MT2*				
12	*Osnadp-me2*	C4 photosynthetic enzymes	Salt and drought stress	EST, Microarray	Muthusamy et al. (2019)
	*Osnadp-me3*				
13	*OsSIPP2C1*	Protein phosphatase 2C	Salt and drought stress	EST, Microarray	Li et al. (2013)
14	*OsFKBP20*	SUMO-	Heat stress	EST	Zhang et al. (2018)
	*OsSce1*	conjugating enzyme and peptidyl prolyl cis-trans isomerase			
15	*OsGLYI6*, *OsGLYI11*	Glyoxalase enzymes	Drought stress	EST, Microarray	Mustafiz et al. (2011)
16	*OsM4*	Meprin and TRAF homology (MATH) domain- containing protein	Salinity and drought	Microarray	Kushwaha et al. (2016)
	*OsMB11*				
17	*OsPP2C*	Protein phosphatase	Salt, cold, and drought	Microarray and Q-PCR	Singh et al. (2010)
	*OsPP2A*				
18	*OsMADS4*, *OsMADS5*, *OsMADS6*, *OsMADS7*, *OsMADS15*	Transcription factors	Aerobic adaptation	RNA-Seq	Phule et al. (2019)
19	*OsSWEET3A*	Sugar transporters	Aerobic adaptation	RNA-Seq	Phule et al. (2019)
20	*OsLEA3*	Late embryogenesis abundant proteins	Drought stress	RNA-Seq	Mangrauthia et al. (2016)
	*OsDREB1A*	Dehydration- responsive element binding			
	*OsRAB16B*	Responsive to ABA			
22	*OsPRX*	Peroxidase precursor	Cold stress	RNA-Seq	Dametto et al. (2015)
	*OsKET*	3-Ketoacyl-CoA synthase			
	*OsAQU*	Aquaporin protein			
	*OsCSLE1*	Cellulose synthase-like family E			
	*OsCDKB2;1*	Cyclin- dependent kinase B2-1			
21	*OsSulfT2.1*, *OsPotT2*	Sulfate transporter	Fluoride susceptibility	RNA-Seq	Banerjee et al. (2020)
		Potassium transporter			
22	*OsMYB-R1*	Transcription factor	Drought stress	RNA-Seq	Tiwari et al. (2020)
23	*OsPhyB*	Phytochrome B	Drought stress	RNA-Seq	Yoo et al. (2017)
24	*OsSweet11*	Sugar transporter	Fluoride tolerance	RNA-Seq	Banerjee et al. (2020)

Two rice cultivars, IR36 (salt-sensitive) and Weiguo (salt tolerant), were used for QTL discovery. This resulted in the identification of *qRSL7*, which have related to relative shoot length (RSL) and situated on chromosome 7 (Li et al., 2018; Mao et al., 2019; Lei et al., 2020). After 36 hours of salt exposure at the budburst stage, RNA sequencing for IR36 and Weiguo identified five differentially upregulated genes in this potential area. *Os0790569700* (*OsSAP16*) a stress-associated protein increased during drought stress, was discovered to have a 1 bp indel difference using qRT-PCR investigation and further deep RNA-Seq (Lei et al., 2020). Using RNA-Seq, it was possible to pinpoint the photosynthesis-related chloroplast genes that were differentially expressed in response to salt, iron, and cold stress. Cold has the most differentially expressed genes (DEGs) related to light and chloroplast responses out of all the genes expressed in each condition (do Amaral et al., 2016; Sunkar and Zhu, 2019). Cold sensitive and cold-tolerant varieties’ comparative transcriptome investigations using RNA-Seq revealed 13,930 and 10,599 DEGs, respectively. Functional classification of these DEGs\s showed that in cold-tolerant variety lipid-binding activity, catalytic and hydrolase activities, photosynthesis, energy and carbohydrate metabolism were enhanced during cold stress, while in susceptible variety absence of photosynthesis related genes, storage products like starch and fatty acids were noticed (Pradhan et al., 2019). These two investigations imply that genes involved in photosynthetic processes play a direct role in the response to cold stress.

Small RNA molecules known as microRNAs (miRNAs) perform crucial regulatory roles in plant development and stress responses (Barrera-figueroa et al., 2012; Sato et al., 2013). The list of rice miRNAs and siRNAs that provide tolerance to diverse abiotic stimuli is summarized in **Table 3**. Four small RNA libraries from the inflorescence of rice plants grown under control conditions and under abiotic stress, conditions like drought, cold, and salt stress were subjected to RNA sequencing using Illumina deep sequencing. This led to the identification of 227 miRNAs belonging to 127 families. These miRNAs’ expression levels has compared, and the results showed that 18, 15, and 10 miRNAs, respectively, were involved in the responses of rice to drought, cold, and salt stress (Barrera-figueroa et al., 2012). Singh and Prasad (2021) reviewed the part played by small interfering RNAs (siRNAs) in the epigenetic modifications of the chromatin region mediated by siRNA-dependent DNA methylation (RdDM) pathways. Plants create dsRNAs that drive DNA methyl transferases to homologous loci for cytosine methylation and engage Pol IV and RNA-dependent RNA polymerase 2 (RDR2) to carry out this task (Matzke and Mosher, 2014). One-third of the methylation loci in Arabidopsis has methylated by this route (Singh and Prasad, 2021). *RDR6-dependent*, *PolII-DCL3-dependent*, *RDR6-DCL3-dependent* and dicer-independent RdDM pathways, as well as other non-canonical RdDM pathways, are known in plants (Liu and He, 2020). When exposed to stress again over the course of succeeding generations, epigenetic markers like DNA methylation serve as stress memories (Wang et al., 2020; Singh et al., 2021; Yuan et al., 2021).

**Table 3:** Examples of miRNAs identified in Rice (Oryza Sativa) under drought, cold and salinity stress.

Stress Condition	Inducible Genes	Responsive miRNAs	Functions	References
**Drought stress**	*SalT (LOC_Os01g24710)* *TIR1* *OsLEA3(LOC_Os05g46480)*	miR393 miR402	Salt/cold tolerance	Zhao et al., 2007; Sunkar and Zhu, 2004; Wang and Long, 2010; Raffaele et al., 2007
**cold stress**	*OsWRKY71* *(LOC_Os02g08440)* *OsMAPK2(LOC_Os03g17700)* *Os05g47550, Os03g42280* *Os01g73250, Os12g16350* *Os03g19380*	miR319, miR389, miR393, miR1320, miR1435 miR1884b, CHY1 CP12-2	Drought/salt tolerance Cold tolerance Pathogen immunity response	Sunkar and Zhu, 2004; Raffaele et al., 2007; Rabbani et al., 2003
**Salinity stress**	*SalT (LOC_Os01g24710)* *OsLEA3 (LOC_Os05g46480)*	miR156, miR158, miR159, miR397, miR398, miR482.2, miR530a, miR1445	Drought tolerance Pathogen immune response Heat stress tolerance	Cui et al., 2015; Rabbani et al., 2003; Arenas-Huertero et al., 2009

During times of temperature stress and at particular stages of plant growth, the RdDM pathway also controls the methylation of transposons and chromatin condensation (Papareddy et al., 2020; Pervaiz et al., 2022). Using RNAi transgenics raises certain biosafety issues because chromatin alteration and transcriptional gene silencing have the potential to cause hereditary modifications that have negative repercussions. Crops cultivated with an appropriate RNAi technique and an assessment of the danger to food safety, however, will help allay biosafety worries. Despite several limitations, sRNAs have a huge promise for improving crops. Key to creating abiotic stress tolerance in crop plants lies in the more recent discoveries of sRNAs and their targets. For the up- and down-regulation of sRNAs, one method is the over-expression and short tandem target mimicry (STTM). Li et al. (2020) created transgenic maize plants for the knockdown of miR166 using STTM technology, and these transgenic plants have demonstrated abiotic stress tolerance. By genetically altering plants to express dsRNA, RNAi has successfully used to improve crop tolerance to a variety of abiotic stresses. The use of artificial miRNAs (amiRNAs) and ta-siRNAs (ata-siRNAs) in the deregulation of target genes is a further intriguing strategy (Cisneros and Carbonell, 2020). Primarily, pre-miRNA or pri-miRNA expression in expression vectors is required to achieve miRNA over-expression. Clustered regularly interspaced short palindromic repeats (CRISPR) and the CRISPR associated protein (Cas), a recent technological advance, have enormous potential for crop development and can be effectively used for editing MIR genes for phenotypic modification. One aspect of miRNAs is that their potential applications as short peptides known as micro peptides (miPEPs), which are translated from pri-miRNA sequences, have not yet been fully investigated (Iborra et al., 2001; Prasad et al., 2020). Thus, in order to close the gap between the growing population and food insecurity, scientists are looking into a variety of technologies with broader applications and acceptance. Compared to traditional target discovery techniques, degradation genome sequencing in conjunction with small RNA sequencing has significant advantages. Cleavage-specific information has provided by degradome sequencing in addition to high-throughput identification of the thousands of targets that miRNAs have cut. Plant stress research using degradome analysis has been successful in both biotic and abiotic environments (Yang et al., 2022; Pervaiz et al., 2022). Using panicles on the day of full emergence, small RNA sequencing has done to identify the miRNAs and understand how they have regulated in Swarnaprabha (SP) rice to tolerate prolonged shade (Tanaka et al., 2019; Yang et al., 2022). Degradome sequencing has applied to the same samples to identify degraded targets. The expression levels of the cleaved targets have examined using microarray and qRT-PCR. Another goal of this work was to identify the SP shade-tolerant phenotypic responses that have controlled by miR-mediated PTGS pathways. Yang et al., 2022 studied that the expression of 191 lncRNAs, 2115 mRNAs, and 32 miRNAs (microRNAs) altered in rice during drought stress. They are essential elements of pathways for protein synthesis, chlorophyll synthesis, hormone signal transduction, and other processes, according to a functional analysis of the data.

The results of (Yang et al., 2022) provide a theoretical framework for additional investigation into the potential function of lncRNA in plant drought resistance as well as new genetic resources for rice breeding to generate drought-resistant crops. MiRNAs are essential for regulating gene expression in plants at various stages of development. For instance, 178 miRNA families have 959 founding members in the rice plant *Oryza sativa* (Pervaiz et al., 2022).

Using high-throughput sequencing (HTS) microarray techniques, gene expression profiling under environmental stresses has carried out (Pervaiz et al., 2022). Zinc finger to cold and drought, *AP2* family to cold and drought, MYB to dehydration, NAC and *bHLH* to drought, ABA and salinity, and *bZIP* to dehydration are some of the members of stress-regulated gene families that have found in crop plants (Wang et al., 2021; Pervaiz et al., 2022). In order to comprehend the role of microRNA, an oligonucleotide microarray has used to regulate the expression profile of rice microRNA against drought stress. Drought was found to be a factor in the development of mir169g along with the mir169 family however, the presence of mir169g was more prominent in roots than in shoots (Tiwari and Rajam, 2022). Therefore, sRNA treatments have the potential to improve crop cultivars’ agronomic features and increase food safety. Understanding the mechanism of post-transcriptional gene regulation in managing plant stress response is made possible by the interaction between miRNAs and their targets (Stephen, 2019; Pervaiz et al., 2022; Yang et al., 2022). RNA-Seq analysis following NaF treatment revealed up regulation of 1303 transcripts and down regulation of 93 transcripts this Increased fluoride levels suppress ABA signaling and biosynthetic pathways, according to expression analysis (Banerjee et al., 2020). However, signaling *via* ABA-independent transcription factors and the gibberellic acid pathway have both activated? When DEGs from the IR-64 and fluoride-tolerant varieties were compared, Khitish found that the fluoride sensitive variety had increased levels of autophagy (Banerjee et al., 2020). Further expression analysis showed that fluoride tolerance was associated with high expression of Sweet11 while fluoride susceptibility was associated with high expression of genes responsible for amino acid transport, monosaccharide transport, and nutrient transport (Banerjee et al., 2020). Root transcriptome analysis of the rice (*Oryza sativa.* L.) after Cd and As treatment revealed that the genes shared by Cd and As stress were involved in signal transduction, trans membrane transport, redox control, stress response, transcriptional regulation and the biosynthesis and metabolism of macromolecules and Sulphur compounds (Zheng et al., 2017; Huang et al., 2019).

## Tools and databases for geneticists and breeders of rice using the genome

Numerous databases have built in order to better organize and make use of the extensive genomic data produced by rice genome studies. Comprehensive databases include a variety of data formats for many different species including rice (*Oryza sativa*. L.), The National Center for Biotechnology’s GenBank database Information (NCBI) is a well-known comprehensive database that offers a sizable collection of biological data and information (https://www.ncbi.nlm.nih.gov/); Ensembl Plant incorporates tools for viewing, mining, and analyzing plant genomes data (https://plants.ensembl.org/); China National Center for Bioinformation’s NGDC- GWH (https://bigd.big.ac.cn/gwh/) is a public repository that holds genome-scale data for a variety of species; rich plant genomics resources may be found at Phytozome (https://phytozome-next.jgi.doe.gov/) and comparative plant resources can be found at Gramene (http://www.gramene.org). A resource for extensive access to details about genome sequencing initiatives is the Genome OnLine Database (GOLD) (https://gold.jgi.doe.gov/). With an emphasis on rice genomic resources including genome sequences, genome annotations and genome variants, several databases are specifically for the grain (**Table 4**). Two well-known databases that offer genome annotation resources for the first rice reference genome Nipponbare are the Rice Annotation Project Database (RAP-DB) and rice Genome Annotation Project (MSU-RGAP) database (Sasaki et al., 2008; Sakai et al., 2013). RAP-genomic DB’s annotations have been updated often. Song et al. created the rice Information Gateway (RIGW) database, which houses multi-omics data covering genomics, transcriptomics, and protein-protein interactions, based on the Indian reference genomes *ZS97* and *MH63* Kawahara et al. (2013). The Molecular Breeding Knowledgebase (MBKBASE) unifies rice germplasm data, population sequencing data, phenotypic data and several other genomics data sets based on Nipponbare and a high-quality indica reference genome, Song et al. (2018). Rice Genome Hub and Information Commons for rice (IC4R) Peng et al. (2018) offer a comprehensive resource for combining multiomics data for rice (*Oryza sativa*. L.) to enable effective epigenomic studies in rice Zhang et al. (2020) created the species-specific epigenomic database eRice (an Epigenomic & Genomic Annotation Database for rice (*Oryza sativa.* L.). Two databases that offer information and resources for rice pan-genome study are rice Pan-genome Browser (RPAN) and rice PanGenome Sun et al. (2017). Oryza Genome is a database of wild Oryza species’ genome diversity that houses genomic materials for the genus Oryza. Twelve rice relatives from the Poaceae family have housed in the Rice Relatives Genomic Data Base (RRGD) Mao et al. (2020). Other databases concentrating on rice genomic variation, gene expression, gene function, and mutations. Rice Functional Genomics & Breeding (RFGB), rice SNP-Seek Database, and rice Variation Map (RiceVar-Map) all give information regarding genomic variation in addition to phenotype, rice cultivars, and functional annotation of variation (Zhao et al., 2015). SnpReady provides Haplotype map (hapmap) SNPs and haplotype information for rice for rice (SR4R) and HapRice, (Yonemaru et al., 2014). Resources for rice transposable elements can be found in the databases rice Transposons Insertion Polymorphism Database (RTRIP) and rice TE Database (RiTE DB) Copetti et al. (2015).

**Table 4:** List of resources for genomic and transcriptome databases of rice is.

Database	Full name		Country	Keywords		Tools	Website	Last update	References
*Genome/pan-genomes*									
RAP-DB	The Rice Annotation Project Database		Japan	Nipponbare; IRGSP-1.0 reference genome; Genome annotation		Tools including keyword search, Blast, ID converter and batch retriever	https://rapdb.dna.affrc.go.jp/	Sep. 2020	Sakai et al. (2013)
MSU-RGAP	Rice Genome Annotation Project		USA	Nipponbare; MSUv7 reference genome; Genome annotation		Rice gene expression, rice gene expression (gene expression plots, gene correlation search within a module or experiment), genome nomenclature, genome facts	http://rice(Oryzasativa.L.).plantbiology.msu.edu/	Feb. 2013	Kawahara et al. (2013)
RIGW	Rice Information Gateway		China	ZS97 reference genome; MH63 reference genome; Omics data; Interaction Data		Orthologous, KEGG/GO enrichment, CRISPR-design etc	http://rice(Oryzasativa.L.).hzau.edu.cn/rice(Oryzasativa.L.)_rs3/	Apr. 2018	Song et al. (2018)
MBKBASE	Molecular Breeding Knowledgebase		China	R498 reference genome; Germplasm information; Population data; Phenotypic data		CustomGT, WebArray, etc	http://www.mbkbase.org/rice(Oryzasativa.L.)	Oct. 2020	Peng et al. (2018)
IC4R	Information Commons for Rice		China	Genome sequences; Genome annotation		HK-TS Gene Finder	http://ic4r.org/	May 2018	Sang et al. (2020)
Rice Genome	–		France	Genomics and genetics		Tools to browse, visualize and search	https://rice(Oryzasativa.L.)-genome-	Jan.	Agret et al. (2020)
Hub				Data		among all data sets available	hub.southgreen.fr/	2019	
ERice (Oryza sativa. L.)	Epigenomic & Genomic Annotation Database for Rice		China	Reference genome 93- 11; DNA methylation; N6- methyldeoxyadenosine (6 mA)		Tools for DNA Methylation analysis and 6 mA AI predictor	http://www.elabcaas.cn/rice(Oryzasativa.L.)/index.html	Jan. 2020	Zhang et al. (2020)
RPAN	Rice Pan- genome Browser		China	Pan-genome browser; 3KRGP			http://cgm.sjtu.edu.cn/3krice(Oryzasativa.L.)db/	Feb. 2018	Sun et al. (2017)
Rice (Oryza sativa. L.)PanGenome			China	Pan-genome; Genome assembly; Genomic variation			http://db.ncgr.ac.cn/Rice(Oryzasativa.L.)PanGenome/	Feb. 2018	Zhao et al. (2018)
OryzaGenome			Japan	Genomic variation; Genus Oryza			http://viewer.shigen.info/oryzagenome2detail/index.xhtml	Oct. 2018	Ohyanagi et al. (2016)
Rice (Oryza sativa. L.)RelativesGD	Rice Relatives Genomic Database		China	Rice relatives; Genome assembly; Genes		Tools for phylogenetic tree build	http://ibi.zju.edu.cn/rice(Oryzasativa.L.)relativesgd/	Apr. 2020	Mao et al. (2020)
***Genomic variation***									
Rice (Oryza sativa. L.)VarMap	Rice Variation Map		China	Genomic variation; Cultivar information		Tools for primer design, haplotype network analysis etc	http://rice(Oryzasativa.L.)varmap.ncpgr.cn/v2/	Jun. 2018	Zhao et al. (2015)
Rice SNP-Seek Database			Philippines Genomic variation; Variety information			Tools for browse 3KRG project related Dat	https://snp-seek.irri.org/	Mar. 2020	Mansueto et al. (2017)
RFGB	Rice Functional Genomics & Breeding		China		Genomic variation; Phenotype information; Germplasm information	3K Grouping (group samples based on population structure of 3K rice (*Oryza sativa.* L.) genome)	http://www.rmbreeding.cn/	Jul. 2019	Wang et al. (2020)
SR4R	SnpReady for Rice		China		Genomic variation based on 4 reference panels; Hapmap SNPs	Tools for basic genotype processing, population diversity analysis and rice (*Oryza sativa*. L.) varieties classification and identification	http://sr4r.ic4r.org/	Jun. 2019	Yan et al. (2020)
HapRice			Japan		SNP; Haplotype	Web tools for finding polymorphic SNPs between any two rice accessions and for primer design to develop cleaved amplified polymorphic sequence markers	http://qtaro.abr.affrc.go.jp/index.html	Aug. 2013	Yonemaru et al. (2014)
RTRIP	Rice Transposons Insertion Polymorphism Database		China		TE polymorphisms; Variety information		http://ibi.zju.edu.cn/Rtrip/index.html	Nov. 2019	Liu et al. (2020)
RiTE DB	Rice TE Database		USA		Repeat sequences; TE		https://www.genome.arizona.edu/cgi-bin/rite/index.cgi	Aug. 2015	Copetti et al. (2015)
***Gene expression***									
Rice FREND	Rice (Oryza sativa. L.) Functionally	Japan			Gene coexpression; Microarray data		https://rice(Oryzasativa.L.)frend.dna.affrc.go.jp/	Sep. 2012	Sato et al. (2013)
Rice XPro	Rice Expression Profile Database	Japan			Gene expression profiles; Microarray data	Exp_blast, ExProFlip	https://rice(Oryzasativa.L.)xpro.dna.affrc.go.jp/		Sato et al. (2013)
RED	Rice Expression Database	China			Gene expression profiles; RNA-Seq	Co-expression and JBrowse	http://expression.ic4r.org/	Aug. 2016	Xia et al. (2017)

***Mutants and mutation***									
RMD	Rice Mutant Database	China			Information of 129,000 rice T-DNA insertion lines; Comprehensive information about mutant phenotypes	Tools for identification of novel genes, regulatory elements etc	http://rmd.ncpgr.cn/	Mar. 2012	Zhang et al (2006)
KitBase	KitaakeX Mutant Database	USA			Fast-neutron-induced mutant population; KitaakeX	Tools including JBrowse, Search and Blast	https://kitbase.ucdavis.edu/home		Li et al. (2017)
MOsDB	The PGSB Oryza sativa Database	Germany			Genomes; Genes; Mutant Information; Expression Profiles	Genome view, Comparative map Viewer	http://pgsb.helmholtz-muenchen.de/plant/rice(Oryzasativa.L.)/index.jsp		Karlowski et al. (2003)
***Bioinformatics tools***									
Rice Galaxy		Philippines				Tools for designing SNP assays, analyzing GWAS, population diversity, rice (*Oryza sativa*. L.)-bacterial pathogen diagnostics, and a suite of published genomic prediction methods	https://galaxy.irri.org/		Juanillas et al. (2019)
Rice Diversity		USA				GWAS Viewer, Genome Browser, Rice (*Oryza sativa*. L.) Sub-population Viewer, Seed Photo Library Viewer, etc	http://rice(Oryzasativa.L.)diversity.org/	Sep. 2017	
FunRice (Oryza sativa. L.)Genes	Functionally Rice Genes	China				An advanced search tool that provides data associated with functionally characterized rice genes such as genes, gene families, keywords, and literature.	https://funrice(Oryzasativa.L.)genes.github.io/		Yao et al. (2018)
DSDecodeM		China				A web tool for rapid decoding of multiple superimposed sequencing chromatograms	http://skl.scau.edu.cn/dsdecode/		Liu et al. (2015)
CRISPR-GE	CRISPR- Genome Editing High- throughput Tracking Of Mutations	China China				A toolkit for CRISPR-based genome editing An online tool to track the mutations with precise percentage for multiple samples and multiple target sites	http://skl.scau.edu.cn/home/ http://www.hi-tom.net/hi-tom/		Xie et al. (2017); Liu et al. (2019)
Hi-TOM						
MMEJ-KO		China				Tool for automatically designing paired guide-RNAs for MMEJ-mediated fragment deletion using CRISPR/Cas genome editing.	http://skl.scau.edu.cn/mmejko/		Xie et al. (2020)
CAFRI-Rice (Oryza sativa. L.)	CRISPR Applicable Functional Redundancy Inspector	The Republic of Korea				CRISPR applicable functional redundancy inspector to accelerate functional genomics in rice (*Oryza sativa*. L.)	http://cafri-rice(Oryzasativa.L.).khu.ac.kr/	Dec. 2019	Hong et al., (2020)

The rice, Functionally Related Gene Expression Network Database (RiceFREND), Rice Expression Profile Database (RiceXPro) and Rice Expression Database (Sato et al., 2013 and Xia et al., 2017) provide Gene expression profiles and co-expressed genes. Data-bases describing rice mutants and mutations include the Rice Mutant Database (RMD), KitaakeX Mutant Database (KitBase), and The PGSB *Oryza sativa* database (MOsDB) Li et al. (2017). eRice offers tools for analysing DNA methylation and a 6 mA AI predictor, while Rice (*Oryza sativa*. L.)VarMap offers tools for analyzing haplotype networks. There are websites other than these databases where you can find web tools made exclusively for research on rice. In addition to a variety of published genomic prediction techniques, Rice Galaxy offers tools for developing SNP tests, assessing GWAS research, population diversity, and diagnosing rice bacterial pathogens Liu et al. (2015). Rice Diversity offers GWAS viewer, Rice Sub-population Viewer, and Seed Photo Library Viewer among other features. Yao et al. (2018) created the comprehensive database fun rice Genes, which contains about 2800 functionally defined rice genes, by fusing publicly accessible data and analyzing papers describing rice functional genomic investigations. A handy, integrated toolkit for accelerating all experimental designs for CRISPR/Cas9/Cpf1-based genome editing and analyzing the resulting mutations in rice and other plants, CRISPR-GE was created by Xie et al, and includes DSDecodeM and MMEJ-KO Xie et al. (2017) and Liu et al. (2019). Using the web Programme (Hi-TOM), it is possible to calculate the precise percentages of gene editing mutations for numerous samples and target sites Xie et al. (2020). CRISPR applicable functional redundancy inspector (CAFRI-Rice) is another gene-editing tool that may be used to locate suitable target genes for editing in order to prevent functional redundancy (Hong et al., 2020)

Over the past year, a number of databases including mRNA sequences acquired from rice have created; a list of sequenced genomes of rice and its relatives summarized in (**Table 5**). A comprehensive mRNA-Seq database for rice called TENOR (Transcriptome ENcyclopedia of rice along with novel genes discovered from mRNA-Seq data, expression profiles, co-expressed genes, and cis-regulatory elements, mRNA sequences from diverse abiotic and hormone treatments are provided. This database is accessible to the public (Kawahara et al., 2016). An integrated gene expression database for rice (Rice Expression Database) (RED) was developed from RNA-Seq data. RED integrates Gene expression patterns from all growth phases and diverse abiotic stressors (Xia et al., 2017). The Rice Environment Coexpression Network (RECoN) is used to analyses abiotic stress responses at the systems level. The novel differential expression profile is helpful in locating groups of tightly co-expressed genes that are functionally characterized and uncharacterized during abiotic stress (Krishnan et al., 2017). Wang et al., to find circular RNA that is responsible for stress circumstances, created CropCircDB, a comprehensive circular RNA resource for crops in response to abiotic stress, in 2019; CropCircDB have developed primarily for rice and maize. Future addition of additional crops is the goal of this database (Wang et al., 2019a; Wang et al., 2019b).

**Table 5:** List of sequenced genomes of rice and its relatives.

Species	Genome type	Genome size (Estimated/assembly)	Predicted gene	Accession	Refrences
*O. sativa (japonica* group*)*	AA (2*n* = 24)	420 Mb/390 Mb	*35 825*	Nipponbare	Kawahara et al., 2013
*O. sativa (indica* group) *O. rufipogon*	AA (2*n* = 24) AA (2*n* = 24)	−−/396 Mb −−/338 Mb	*38 729* *37 071*	R498 W1943	Du et al., 2017; Stein et al., 2018
*O. nivara*	AA (2*n* = 24)	−−/338 Mb	*36 313*	IRGC100897	Stein et al., 2018
*O. glaberrima*	AA (2*n* = 24)	−−/316 Mb	*33 164*	CG14	Wang et al., 2014
*O. barthii*	AA (2*n* = 24)	−−/308 Mb	*34 575*	IRGC105608	Stein et al., 2018
*O. glumaepatula*	AA (2*n* = 24)	−−/373 Mb	*38 149*	GEN1233_2	Stein et al., 2018
*O. meridionalis*	AA (2*n* = 24)	−−/336 Mb	*34 897*	W2112	Stein et al., 2018
*O. puntaca*	BB (2*n* = 24)	−−/394 Mb	*31 762*	IRGC105690	Stein et al., 2018
*O. brachyantha*	FF (2*n* = 24)	297 Mb/261 Mb	*32 037*	IRGC101232	Chen et al., 2013
*Leersia perrieri*	−−/(2*n* = 24)	−−/267 Mb	*29 078*	IRGC105164	Stein et al., 2018
*Zizania latifolia*	−−/(2*n* = 34)	594 Mb/604 Mb	*43 703*	HSD2	Guo et al., 2015

The term “single-cell sequencing technologies” refers to the sequencing of a single cell’s genome and/or transcriptome in order to obtain genomic, transcriptomic, or other multi-omics information with the goal of learning about the differences between different cell populations and the relationships between them throughout time (Tang et al., 2019). The data produced by sequencing could be extremely heterogeneous, making bioinformatics analysis difficult, depending on the tissues used. Recent research on the biology of rice has used single-cell sequencing. Han et al. studied the allelic expression patterns in the mesophyll cells of the 93-11 and Nipponbare inbred lines, as well as of their F1 reciprocal hybrids, in 2017 using a bioinformatics technique based on single-cell RNA-seq data from rice (Han et al., 2017). A common RNA-seq variant analysis workflow has used to find SNPs between indica and japonica rice using RNA-seq data from rice mesophyll cells. A bioinformatics approach has built to categorize genes into biallelic, monoallelic and silent genes by combining the information from SNPs and SNP-covering reads. An excellent opportunity to look into the causes and prevalence of monoallelic gene expression in plant cells have provided by the development of a single-cell RNA-seq bioinformatics analysis procedure in this study. To compare three-dimensional (3D) chromatin organization and dynamics before and after rice fertilization, Zhou et al. created a high-resolution *in situ* Hi-C approach and examined individual nuclei isolated from rice eggs, sperms, unicellular zygotes, and shoot mesophyll cells in a different study based on single-cell sequencing and Hi-C (Zhou et al., 2019). Their findings offered a spatial chromatin basis for zygotic genome activation and epigenetic control in rice as well as distinct 3D genome characteristics of rice gametes and the unicellular zygote. Recently, the cell biology of rice plants growing in varied environments has studied using single-cell sequencing. (Wang et al., 2020) used a bioinformatics pipeline based on single-cell RNA sequencing to identify the main cell types and recreate their developmental trajectories. According to their research, abiotic stressors have an impact on cell-type-specific gene expression as well as the physical dimensions of cells and the makeup of cell populations.

## Conclusion

The world’s main crop is rice (*Oryza sativa.* L.). Production of rice is impacted by agricultural difficulties like biotic and abiotic stressors. In order to combat these pressures and the growing global population, rice productivity must increase. Rice productivity has negatively affected by abiotic factors such as drought, cold, salt, and heavy metal stress. Two main tactics have used to combat the detrimental impact of these undesirable effects: (2) crop breeding, and (1) crop management techniques. Multi-omics methods like genomics, transcriptomics, proteomics and metabolomics are currently supporting crop-breeding technologies for the discovery of new genes, protein regulatory networks and functional study of existing genes. Among these omics methods, genomics and transcriptomics greatly contribute by identifying genes that are responsive to abiotic stress, creation of numerous markers and QTLs for marker-assisted breeding using genome-wide analyses of different rice accessions. New genes, transcription factors, and non-coding RNAs implicated in the response to abiotic stress has found by combining transcriptome analysis with next-generation sequencing (NGS). In the past few decades, numerous genes in rice has discovered, and numerous elite cultivars have been created in rice using gene pyramiding, transgenic technology and gene editing., however functional genes must be investigated. The study of numerous orphan crops, halophytes, thermophytes, and other plants living in extreme environments can help identify new target genes in rice that respond to abiotic stress, Less undesirable genes would result from successful breeding, and during natural selection, many undesirable traits are passed on to future generations along with desirable ones. These genes must be located and their structural makeup examined in order to eliminate. Integrated omics techniques could be used to apply all of these.

## Prospective

Genomics and transcriptome technology can quickly identify the necessary defense factors against stress and reveal the connections between metabolic pathways, signal transduction, and defense response, which are crucial for enhancing plant stress resistance and comprehending the mechanism underlying plant stress resistance. Plants to respond to stress use sophisticated metabolic networks and a wide variety of intricate cellular, molecular and physiological processes. The understanding of plant antistress is still limited, and more research is required to determine the synergistic effect of these pathways or other associations. Although several genes related to the antistress metabolic pathway in plants have cloned and their molecular mechanisms have been gradually been elucidated, the understanding of plant antistress is still limited. There may be a cross-interaction when plants are able to withstand different pressures in the natural environment. It is important to comprehend the distinct and shared signaling and metabolic pathways that have found in plants. The research of plant resistance genes centers on the signal transduction and metabolic pathways that are unique to and shared by different plant species. We must employ a range of techniques in this study process to fully and to completely depict the molecular mechanism of plant resistance.

## Author contributions

The author contributed to the planning the work, to the writing of the manuscript, and to designing the figure.

## Acknowledgment

It is acknowledged that the Emirates Professor P. Stephen Baenziger, Department of genetics and Plant Breeding University of Nebraska Lincoln-USA provided valuable guidance and suggestions during manuscript writing and IT department university of Nebraska USA are grateful for making e-resources available to author

## Conflict of interest

The author declares that the research was conducted in the absence of any commercial or financial relationships that could be construed as a potential conflict of interest.

## Publisher’s note

All claims expressed in this article are solely those of the authors and do not necessarily represent those of their affiliated organizations, or those of the publisher, the editors and the reviewers. Any product that may be evaluated in this article, or claim that may be made by its manufacturer, is not guaranteed or endorsed by the publisher.
